# Discoloration by Topical Use of Nitrate of Silver

**Published:** 1853-01

**Authors:** 


*Discoloration by topical use of Nitrate of Silver.*—At a meeting of the New Hampshire State Medical Society, held in June last, a clergyman was introduced to the society, whose skin had been deeply stained by the topical application of nitrate of silver. The change of color did not appear until after the protracted and profuse application of it. The relief experienced from its use was so great that, though cautioned as to its effects, he continued to use it, and said, "if it is necessary for me to use it to enable me to speak, I shall do so, though it makes me as black as a hat." He is otherwise in good health. None of the means used have had any effect in removing the color.—*New York Medical Times.*

